# IILLS: predicting virus-receptor interactions based on similarity and semi-supervised learning

**DOI:** 10.1186/s12859-019-3278-3

**Published:** 2019-12-27

**Authors:** Cheng Yan, Guihua Duan, Fang-Xiang Wu, Jianxin Wang

**Affiliations:** 1 School of Computer Science and Engineering, Central South University, 932 South Lushan Rd, ChangSha, 410083 China; 2 School of Computer and Information,Qiannan Normal University for Nationalities, Longshan Road, DuYun, 558000 China; 3 Biomedical Engineering and Department of Mechanical Engineering, University of Saskatchewan, Saskatoon, SKS7N5A9 Canada

**Keywords:** Virus-receptor interaction, Similarity, Semi-supervised learning, Laplacian regularized least squares classifier, Gaussian interaction profile (GIP) kernel

## Abstract

**Background:**

Viral infectious diseases are the serious threat for human health. The receptor-binding is the first step for the viral infection of hosts. To more effectively treat human viral infectious diseases, the hidden virus-receptor interactions must be discovered. However, current computational methods for predicting virus-receptor interactions are limited.

**Result:**

In this study, we propose a new computational method (IILLS) to predict virus-receptor interactions based on Initial Interaction scores method via the neighbors and the Laplacian regularized Least Square algorithm. IILLS integrates the known virus-receptor interactions and amino acid sequences of receptors. The similarity of viruses is calculated by the Gaussian Interaction Profile (GIP) kernel. On the other hand, we also compute the receptor GIP similarity and the receptor sequence similarity. Then the sequence similarity is used as the final similarity of receptors according to the prediction results. The 10-fold cross validation (10CV) and leave one out cross validation (LOOCV) are used to assess the prediction performance of our method. We also compare our method with other three competing methods (BRWH, LapRLS, CMF).

**Conlusion:**

The experiment results show that IILLS achieves the AUC values of 0.8675 and 0.9061 with the 10-fold cross validation and leave-one-out cross validation (LOOCV), respectively, which illustrates that IILLS is superior to the competing methods. In addition, the case studies also further indicate that the IILLS method is effective for the virus-receptor interaction prediction.

## Background

Viruses are the most abundant biological entities on the planet and widely distributed in organs of living organisms and environments [1, 2]. In particular, they are an important part of the human microbiome which is closely related with human health and diseases [3]. Actually, hundreds of human diseases were resulted from viruses [4], such as Ebola virus (EBOV) [5], Zika virus [6], American Machupo virus (MACV), Guanarito virus (GTOV), Sabia virus (SABV), Junin virus (JUNV), and so on [7]. In marine environments, viruses can kill up to 40% of the standing stock of prokaryotes daily [8]. In addition, the cellular and physiological changes in the host cells can be caused by virus infections, such as altering genomic sequences and dysfunctioning their hosts [9, 10].

When viruses contact the surface of host cells, the virus process starts [11]. In general, the receptor-binding is considered as the first step for the viral infection of host cells [12]. The specificity and affinity are the main factors that viruses can use diverse types of molecules to attach to and enter into cells [13]. With the development of high-throughput technologies, many studies indicate that some molecules including proteins are the receptor of viruses [14], such as carbohydrates and lipids [15]. Furthermore, the virus-receptor interaction is also an dynamic process, as it can evolve over the course of an infection while virus variants with distinct receptor-binding specificity and tropism can appear [13]. In order to help understand the interaction mechanism between viruses and receptors, a database (called viralReceptor) with mammalian virus-receptor interactions has been constructed by Zhang et.al [16]. ViralReceptor consists of 128 viral species or sub-species, 119 receptors of mammalian and 268 interaction pairs between them. In addition, the structural and functional analysis of receptors also further provide the theoretic basis to discover new virus-receptor interactions, which include protein domains, higher level of N-glycosylation, higher ratio of self-interaction, and so on [16].

In this study, we propose a computational method (IILLS) based on Initial Interaction scores method via the neighbors and Laplacian regularized Least Square algorithm (a semi-supervised learning method), to predict virus-receptor interactions. IILLS integrates the known virus-receptor interactions and amino acid sequences of receptors to compute similarities of viruses and receptors. Then IILLS uses the Laplacian regularized Least Square algorithm and initial interaction scores based on the neighbors to construct the computational model. We conduct the 10-fold cross validation (10CV) and leave one out cross validation (LOOCV) to assess the prediction performance of IILLS and compare it with other three methods. The prediction performance of IILLS is best in terms of AUC (the area under of ROC curve) as its AUC values are 0.8675 and 0.9061 with 10CV and LOOCV, respectively. The evaluation results of case study also show that IILLS is an effective virus-receptor prediction method.

We also provide IILLS, via a web server, to predict virus-receptor interactions. The input of this web server is a receptor amino acid sequence or a txt file with multiple sequences in the FASTA format. The prediction result will be displayed after submission when uploading a sequence. However, the prediction results of the txt file of sequences is sent by the email with link page. Therefore, when uploading a sequence file, an email address should be provided. In addition, a job ID is assigned after one submission. According to job ID, the user can also obtain the prediction result from web server.

## Methods

### Materials

**Fig. 1:**
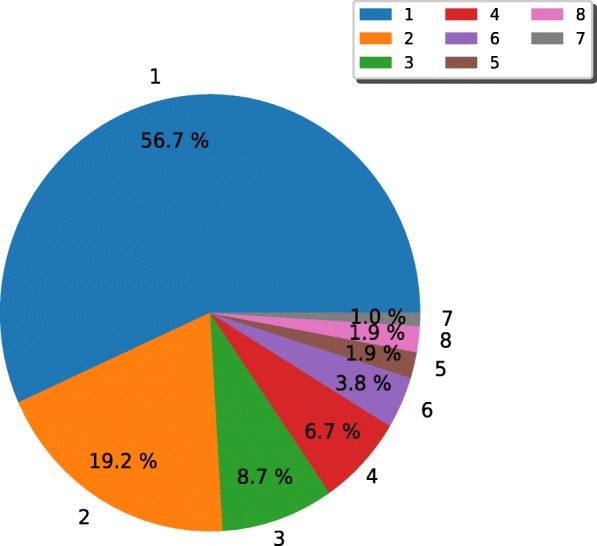
The proportion of viruses’ node degree (Total =104)

**Fig. 2:**
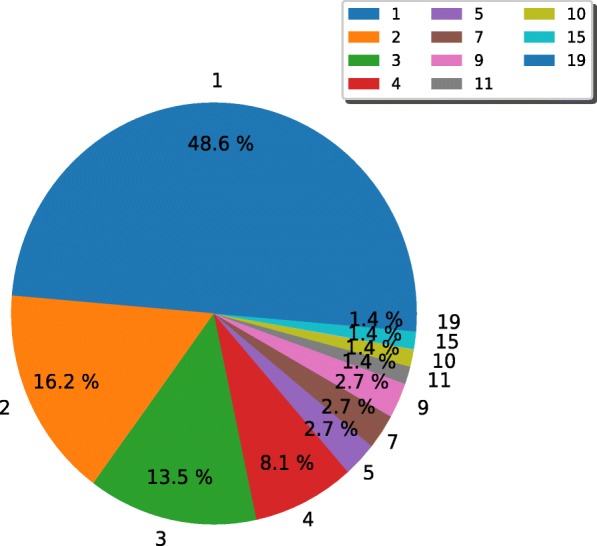
The proportion of receptors’ node degree (Total =74)

We download the known mammalian virus-receptor interactions from viralReceptor database. Then we further extract human virus-receptor interactions as the benchmark dataset. It includes 104 virus species or sub-species, 74 receptors and 211 interaction pairs between viruses and receptors. The detail node degree distributions of viruses and receptors in this standard virus-receptor interaction network are also described in Figs. 1 and 2. The degree of a node is the number of edges which have this node as an endvertex in the virus-receptor interaction network. Each color represents the proportion of viruses (receptors) which have the same node degree. In Fig. 1, the node degrees of 104 virus range from 1 to 8, respectively. Their distribution proportion are 56.7%, 19.2%, 8.7%, 6.7%, 1.9%, 3.8%, 1.0% and 1.9%, respectively. In Fig. 2, each color represents the proportion of receptors with the same node degree. For example, the red color represents that 8.1% of all receptors have the node degree of 4.

### Similarity of viruses

Based on the assumption that similar viruses exhibit similar interaction profiles with receptors [17–20], we used the Gaussian Interaction Profile (GIP) similarity to measure the virus similarity. Let $\phantom {\dot {i}\!}V=\{v_{1},v_{2},...,v_{N_{v}}\}$ be the set of *N*_*v*_ viruses, $\phantom {\dot {i}\!}P=\{p_{1},p_{2},...,p_{N_{p}}\}$ be the set of *N*_*p*_ receptors, and $\phantom {\dot {i}\!}Y \in R^{N_{v} \times N_{p}}$ be the adjacency matrix of the bipartite graph to describe known virus and receptor associations. When the virus *v*_*i*_ and receptor *p*_*j*_ have a known interaction, the value of *y*_*ij*_ is 1 and otherwise 0. The GIP similarity of viruses *v*_1_ and *v*_2_ can be computed as follows:

1 $$\begin{array}{@{}rcl@{}}  {S_{v}(v_{1},v_{2})} = {G_{v}(v_{1},v_{2})} = exp\left(-\gamma_{v} {||{yv}_{1}-{yv}_{2}||}^{2}\right), \end{array} $$

2 $$\begin{array}{@{}rcl@{}}  \gamma_{v} = \gamma{^,_{v}}/\left(\frac{1}{N_{v}}\sum\limits_{i=1}^{N_{v}}{||{yv}_{i}||}^{2}\right), \end{array} $$

in which $\phantom {\dot {i}\!}{yv}_{1}=\{y_{11},y_{12},...,y_{{1}{N_{p}}}\}$ and $\phantom {\dot {i}\!}{yv}_{2}=\{y_{21},y_{22},...,y_{{2}{N_{p}}}\}$ are the interaction profiles of virus *v*_1_ and virus *v*_2_, respectively. The parameter *γ*_*v*_ is used to regulate the kernel bandwidth. We can set the value of bandwidth parameter *γv*, by the cross validation. In this study, the parameter *γv*, is set to be 1 according to previous successful studies [17, 21, 22] and the influence analysis of prediction performance of parameter *γv*, by the 10-fold cross validation.

### Similarity of receptors

In this study, we take two methods to measure the receptor similarity, which include the GIP similarity and the amino acid sequence similarity. The GIP similarity of receptors is also computed by the known interactions of receptors. Specifically, for receptors *p*_1_ and *p*_2_, their GIP similarity can be calculated as follows:

3 $$\begin{array}{@{}rcl@{}}  {G_{p}(p_{1},p_{2})} = exp\left(-\gamma_{p} {||{yp}_{1}-{yp}_{2}||}^{2}\right), \end{array} $$

4 $$\begin{array}{@{}rcl@{}}  \gamma_{p} = \gamma{^,_{p}}/\left(\frac{1}{N_{p}}\sum\limits_{i=1}^{N_{p}}{||{yp}_{i}||}^{2}\right), \end{array} $$

in which ${yp}_{1}=\{y_{11},y_{21},...,y_{{N_{v}}{1}}\}^{T}$ is the interaction profile of receptor *p*_1_ while ${yp}_{2}=\{y_{12},y_{22},...,y_{{N_{v}}{2}}\}^{T}$ is the interaction profile of receptor *p*_2_. Furthermore, the parameter *γ*_*p*_ is also used to control the kernel bandwidth and the parameter *γp*, is also set to be 1.

In addition, we compute the sequence similarity between receptors. First, we download the amino acid sequences of receptors from the KEGG GENE database [23]. The receptor sequence similarity is computed by their normalized Smith-Waterman score [24, 25]. For receptors *p*_1_ and *p*_2_, the sequence similarity can be calculated as follows:

5 $$\begin{array}{@{}rcl@{}}  {G_{s}(p_{1},p_{2})} = SW(p_{1},p_{2})/{\sqrt{SW(p_{1},p_{1})}\sqrt{SW(p_{2},p_{2})}}, \end{array} $$

in which *SW*(*p*_1_,*p*_2_) is the original Smith-Waterman score between receptor *p*_1_ and receptor *p*_2_.

Based on the GIP similarity and the sequence similarity of receptors, we construct the final similarity of receptors *S*_*p*_ as follows:

6 $$\begin{array}{@{}rcl@{}}  S_{p} = \alpha*G_{p}+(1-\alpha)*G_{s}, 0 \leq \alpha \leq 1.0 \end{array} $$

where *α* is the weight parameter.

### Initialized interaction profiles for new viruses and receptors

The quality of known virus-receptors has important impact on the performance of prediction method. In this study, we want to set the initialized interaction scores for viruses (receptors) which have no known interaction with receptors (viruses). Inspired by the KNN method, we take the interaction profiles of all neighbors into consideration, which have known interactions. For example, the initial interaction profile between a new virus *v*_*i*_ and receptor *p*_*j*_ can be calculated as follows:

7 $$ y(v_{i},p_{j}) = \frac{\sum\limits_{l=1}^{N_{v}} S{^{(il)}_{v}}y_{lj}}{\sum\limits_{l=1}^{N_{v}} S{^{(il)}_{v}}}   $$

in which $S{^{(il)}_{v}}$ is the GIP similarity between viruses *v*_*i*_ and *v*_*l*_.

Similarly, we also apply the same model to calculate the interaction profiles of new receptor. Specifically, the initial interaction profile between virus *v*_*i*_ and a new receptor *p*_*j*_ can be calculated as follows:

8 $$ y(v_{i},p_{j}) = \frac{\sum\limits_{l=1}^{N_{p}} S{^{(jl)}_{p}}y_{il}}{\sum\limits_{l=1}^{N_{p}} S{^{(jl)}_{p}}}   $$

in which $S{^{(jl)}_{p}}$ is the final similarity between receptors *p*_*j*_ and *p*_*l*_.

### Laplacian regularized least square for virus-receptor interaction prediction

Inspired by successful applications of Laplacian regularized Least Square (LapRLS) model in predicting drug-target interactions [26–28], we adopt the LapRLS model to predict virus-receptor interactions. After obtaining the similarity matrices, we construct the normalized Laplacian matrices for viruses and receptors as follows:

9 $$  L^{v} = (D^{v})^{-1/2}(D^{v}-S_{v})(D^{v})^{-1/2},  $$

10 $$  L^{p} = (D^{p})^{-1/2}(D^{p}-S_{p})(D^{p})^{-1/2},  $$

where the matrix *D*^*v*^ is the diagonal matrix whose element *D*^*v*^(*i*,*i*) is calculated by the sum of row *i* of the virus similarity matrix *S*_*v*_. Similarly, the matrix *D*^*p*^ is calculated based on the receptor similarity matrix *S*_*p*_.

For viruses and receptors, prediction matrixes *F*_*v*_ and *F*_*p*_ are respectively calculated from the LapRLS model by minimizing the cost functions as follows:

11 $$  F{_{v}^{*}} = \underset{F_{v}}{arg \ min} {\left[ ||Y-F_{v}||{_{F}^{2}} + \beta_{v} tr\left({F{_{v}^{T}}}{L^{v}}{F_{v}}\right)\right]},  $$

12 $$  F{_{p}^{*}} = \underset{F_{p}}{arg \ min} {\left[ ||Y-F_{p}||{_{F}^{2}} + \beta_{p} tr\left({F{_{p}^{T}}}{L^{p}}{F_{p}}\right)\right]},  $$

in which *tr*(.) is the trace of a matrix, *Y* is the adjacency matrix of the known virus-receptor interactions, *L*_*v*_ and *L*_*p*_ are the normalized Laplacian matrices of virus similarity and receptor similarity, and ||.||_*F*_ is the Frobenius norm. *β*_*v*_ and *β*_*p*_ are the trade-off parameters and are set to be 1. According to previous studies [29], the computation model can be solved by:

13 $$  F{_{v}^{*}} = S^{v}(S^{v}+\beta_{v}L^{v}S^{v})^{-1}Y,  $$

14 $$  F{_{p}^{*}} = S^{p}(S^{p}+\beta_{p}L^{p}S^{p})^{-1}Y^{T},  $$

Finally, we obtain the virus-receptor interaction prediction matrix *F*^∗^ by the mean of results of viruses and receptors:

15 $$  F^{*} = \left({F{_{v}^{*}}+(F{_{p}^{*}})^{T}}\right)/{2}.  $$

## Results

### Performance evaluation

In order to assess the prediction performance of IILLS, we conduct the 10CV and LOOCV. The AUC is the metric to evaluate the prediction performance. We compare our method with other three methods: BRWH [30], LapRLS [26] and CMF [31].

### Comparison with other methods

**Fig. 3:**
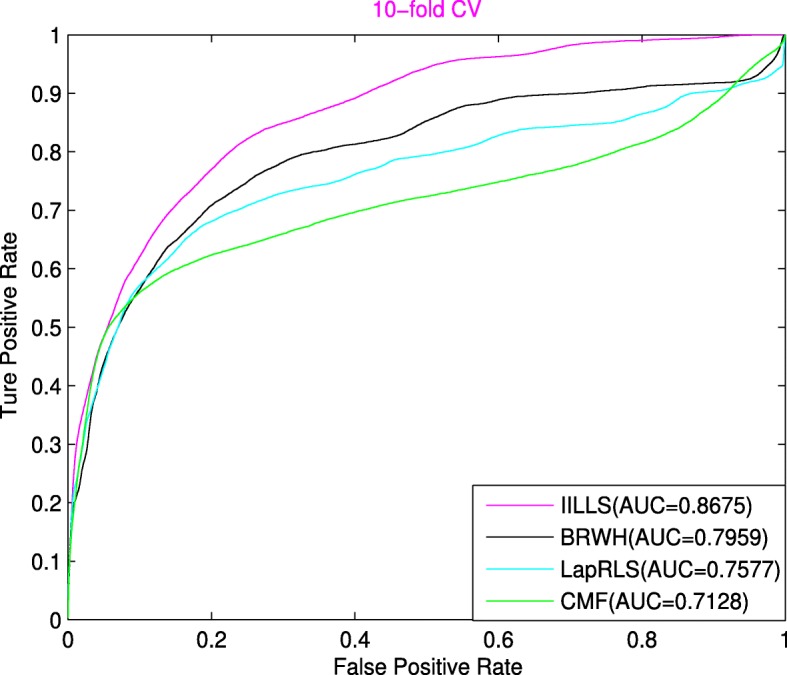
The ROC curves of four methods in 10CV

Figure 3 shows the prediction performance of four methods in 10CV. Compared with other methods (BRWH: 0.7959, LapRLS: 0.7577, CMF: 0.7128), IILLS achieves the best prediction performance with the AUC value of 0.8675.

**Fig. 4:**
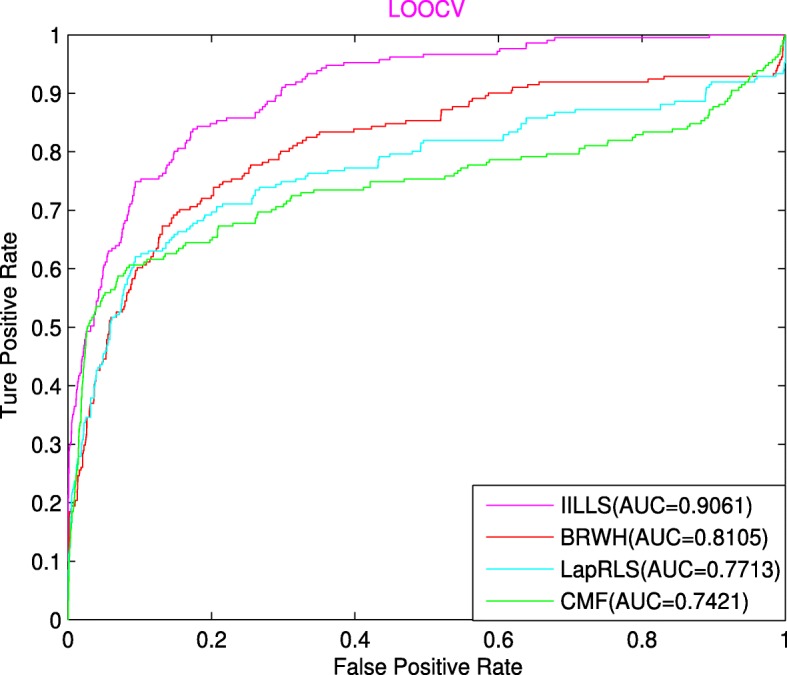
The ROC curves of four methods in LOOCV

Figure 4 also shows that IILLS is superior to other methods in terms of AUC values (IILLS: 0.9061, BRWH: 0.8105, LapRLS: 0.7713, CMF: 0.7421). These experiment results illustrate that IILLS can obtain the better prediction performance.

### Analyzing receptor similarity

In this study, we also analyze the receptor similarity based on the GIP similarity and sequence similarity in terms of the influences of prediction performance of parameter *α* in our method. We conduct 10CV and LOOCV to compute the prediction performance.

Table 1 shows the 10CV prediction performances of various parameter values of *α* ranging from 0 to 1.0 with the increment of 0.1. We can see from Table 1 that our method obtains the best prediction performance in 10CV when only using sequence similarity (*α*=0). The AUC value of our method has a slightly descending trend when *α* ranges from 0 to 1.0.

**Table 1:** The 10CV prediction performances of various parameter values of *α* ranging from 0 to 1.0 with the increment of 0.1, the best result is in the bold face

*α*	0	0.1	0.2	0.3	0.4
AUC	**0.8675**	0.8611	0.8544	0.8500	0.8475
0.5	0.6	0.7	0.8	0.9	1.0
0.8464	0.8425	0.8417	0.8376	0.8327	0.8242

Table 2 shows the LOOCV prediction performances of various parameter values of *α* ranging from 0 to 1.0 with the increment of 0.1. We can see from Table 2 that our method also obtains the best prediction performance in LOOCV when only using sequence similarity (*α*=0). The AUC value of our method has also a slightly descending trend when *α* ranges from 0 to 1.0. Therefore, we set the *α* to be 0 in this study.

**Table 2:** The LOOCV prediction performances of various parameter values of *α* ranging from 0 to 1.0 with the increment of 0.1, the best result is in the bold face

*α*	0	0.1	0.2	0.3	0.4
AUC	**0.9061**	0.8975	0.8935	0.8905	0.8885
0.5	0.6	0.7	0.8	0.9	1.0
0.8865	0.8846	0.8828	0.8806	0.8779	0.8724

In addition, we also provide the ROC of our method on different values of parameter *α* in three cases. The first only uses the sequence similarity of receptors (*α*=0). The second only uses the GIP similarity of receptors (*α*=1.0). The third is with the mean of GIP similarity and sequence similarity of receptors (*α*=0.5).

**Fig. 5:**
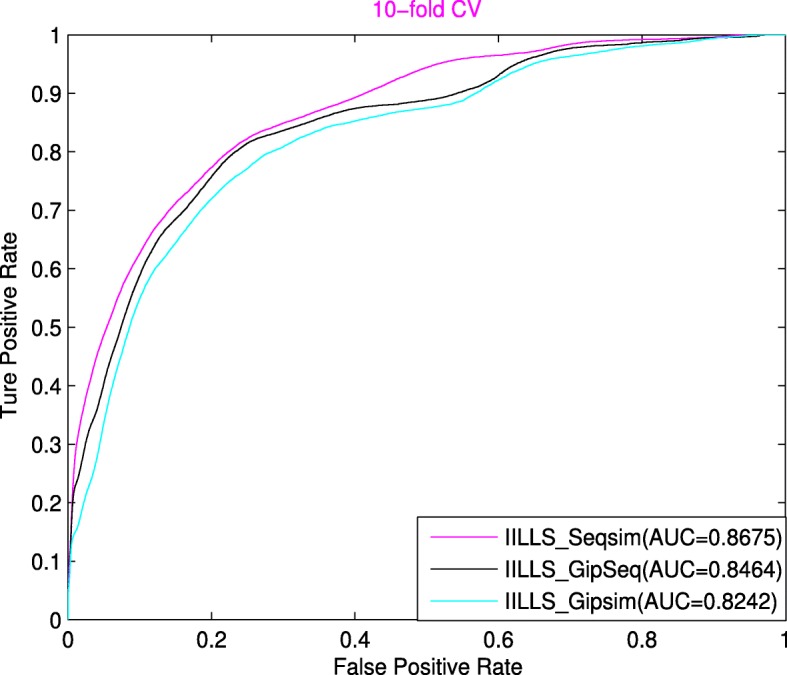
The ROC curves of IILLS under three different receptor similarities in 10CV

**Fig. 6:**
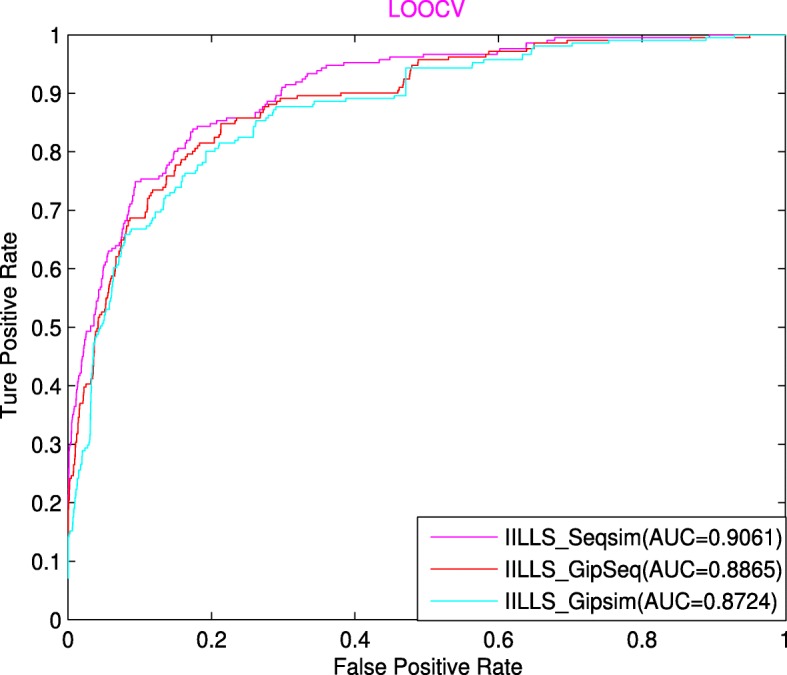
The ROC curves of IILLS under three different receptor similarities in LOOCV

Figures 5 and 6 show the prediction performances of IILLS under three different receptor similarities in 10CV and LOOCV, respectively. We can also see from Figs. 5 and 6 that IILLS achieves the best prediction performance when only using the sequence similarity.

### Parameter analysis for *γv*,

In this section, we analyze parameters *γv*,. In addition, by considering the effect of parameter *γv*, is similar to the effect of parameter *γp*,, we set *γp*,=*γv*,. When only using the sequence similarity, Table 3 shows the 10CV prediction performances of value set (0.25, 0.5, 1, 2, 4) of parameter *γv*,. We can see from Table 3 that our method obtains best prediction performance in 10CV when *γv*, is set to be 2. The AUC value under setting *γv*,=2 is slightly better than the AUC value when *γv*,=1. Therefore, we also simply set the *γv*,=1 as the default value based on the previous successful studies and experiment results of 10CV.

**Table 3:** The 10CV prediction performances of various parameter values of *γv*,, the best result is in the bold face

*γv*,	0.25	0.5	1	2	4
AUC	0.8550	0.8608	0.8675	**0.8700**	0.8434

### Case studies

In order to further evaluate the prediction performance of IILLS in applications, we analyze the prediction ability of our method in discovering new virus-receptor interactions. The extracted human virus-receptor interactions are used as the benchmark datasets. Table 4 shows the validation results of top 10 virus-receptor interactions which are predicted by IILLS. We can see from Table 4 that 5 of 10 predicted associations are validated by previous studies. C-type lectin domain family 4 member M (CLEC4M, also called L-SIGN or CD209L) is equipped with a carbohydrate recognition domain (CRD) that mediates the recognition of fucose and high-mannose glycans in a Ca2+-dependent manner, these carbohydrate structures can be found in multiple pathogens, such as Lassa virus, Ebola virus, among others [32, 33]. The CD209 is also the receptor of known SARS-CoV, human coronaviruses and 229E, although the disease caused by SARS-CoV differs from the diseases caused by the known human coronaviruses and 229E [34]. L-SIGN (also called DC-SIGN) is related to CLEC4M and is a C-type lectin involved in both innate and adaptive immunity, they are known to bind to multiple pathogens and function as cellular receptors for various viruses, such as Dengue virus [35]. Rift Valley fever virus (RVFV) goes through L-SIGN to infect cells expressing the lectin ectopically [32, 36]. The phleboviruses, such as Uukuniemi virus (UUKV), can exploit L-SIGN for infection [32, 36].

**Table 4:** The validated result of top 10 predicted virus-receptor interactions

Rank	Virus	Receptor	References
1	Lymphocytic choriomeningitis mammarenavirus (LCMV)	C-type lectin domain family 4 member M(CLEC4M, L-SIGN)	Unknown
2	Lassa mammarenavirus	C-type lectin domain family 4 member M	Garcia-Vallejo et al, (2015) and Sakuntabhai et al., (2005)
3	Human coronavirus 229E (229E)	CD209 molecule (CD209)	Lo et al., (2006)
4	Dengue virus	C-type lectin domain family 4 member M	Li et al., (2012)
5	Rift Valley fever virus	C-type lectin domain family 4 member M	Lger et al., (2016), and Sakuntabhai et al., (2005)
6	Uukuniemi virus	C-type lectin domain family 4 member M	Lger et al., (2016), and Sakuntabhai et al., (2005)
7	Human immunodeficiency virus 2	C-type lectin domain family 4 member M	Unknown
8	Human alphaherpesvirus 1	integrin subunit beta 3 (beta 3 integrin)	Unknown
9	Coxsackievirus A9 (CAV9)	integrin subunit beta 1	Unknown
10	Human betaherpesvirus 5	integrin subunit beta 6	Unknown

## Discussion

With the development of high-through sequencing technology and microbiology, many studies have evidenced that microbes have key impacts on health body and human diseases. Furthermore, the viruses are an important part of the human microbiomes, and are also the direct origin of infectious diseases, such as Sabia virus and so on. The receptor-binding is the first step for viral infection of host cells. Therefore, in order to systematically understand the mechanisms between virus and receptor and improve the diagnosis and treatment of infectious diseases, it need develop effective methods to identify new virus-receptor interactions.

## Conclusion

In this study, we develop a computational method (IILLS) to predict virus-receptor interactions of human with known virus-receptor interactions and the amino acid sequence of receptors. Firstly, IILLS computes the virus similarity by GIP kernel. Then we also calculate the receptor GIP kernel similarity and the receptor sequence similarity. The final receptor similarity is constructed by the sequence similarity based on the experiment results. IILLS uses the Laplacian regularized Least Square (LapRLS) model to predict the potential virus-disease interactions. It further improves the prediction performance by adding an initial interaction scores process for new viruses and receptors. In terms of AUC with 10CV and LOOCV, IILLS can achieves better prediction performance than other three competing methods. The case studies also show that IILLS can effectively predict virus-receptor interactions, and also help control the virus infectious diseases in the future.

However, there still exist some limitations in IILLS. On the one hand, the virus similarity is calculated by the GIP kernel with known virus-receptor interactions. We should consider more relevant biological network information, such as sequence information. In addition, other integration methods of receptor similarity also should be considered in the future. Finally, other latest matrix factorization methods also should be considered, such as DNRLMF-MDA [37], DRRS [38], SIMCLDA[39] and BNNR [40]. Therefore, we would like to develop a more effective method for predicting virus-receptor interactions by addressing the above limitations in the future.

## Data Availability

The web server of IILLS method is available at http://bioinformatics.csu.edu.cn/IILLS.

## Abbreviations

10CV: 10-fold cross validation
AUC: Area under the receiver operating curve
BRWH: Bi-random walk on a heterogeneous network
CMF: Collaborative matrix factorization
GIP: Gaussian interaction profile
KNN: K-nearest neighbors
LapRLS: Laplacian regularized least squares classifier
SW: Normalized Smith-Waterman score

## References

[1] Minot S, Sinha R, Chen J, Li H, Keilbaugh SA, Wu GD, Lewis JD, Bushman FD (2011). The human gut virome: inter-individual variation and dynamic response to diet. Genome Res.

[2] Paez-Espino D, Eloe-Fadrosh EA, Pavlopoulos GA, Thomas AD, Huntemann M, Mikhailova N, Rubin E, Ivanova NN, Kyrpides NC (2016). Uncovering earth’s virome. Nature.

[3] Wigington CH, Sonderegger D, Brussaard CP, Buchan A, Finke JF, Fuhrman JA, Lennon JT, Middelboe M, Suttle CA, Stock C (2016). Re-examination of the relationship between marine virus and microbial cell abundances. Nat Microbiol.

[4] Geoghegan JL, Senior AM, Di Giallonardo F, Holmes EC (2016). Virological factors that increase the transmissibility of emerging human viruses. Proc Natl Acad Sci.

[5] Maganga GD, Kapetshi J, Berthet N, Kebela Ilunga B, Kabange F, Mbala Kingebeni P, Mondonge V, Muyembe J-JT, Bertherat E, Briand S (2014). Ebola virus disease in the democratic republic of congo. New England J Med.

[6] Mlakar J, Korva M, Tul N, Popović M, Poljšak-Prijatelj M, Mraz J, Kolenc M, Resman Rus K, Vesnaver Vipotnik T, Fabjan Vodušek V (2016). Zika virus associated with microcephaly. New England J Med.

[7] Moraz M-L, Kunz S (2011). Pathogenesis of arenavirus hemorrhagic fevers. Expert Rev Anti-Infect Ther.

[8] Suttle CA (2007). Marine viruses-major players in the global ecosystem. Nat Rev Microbiol.

[9] Qin N, Yang F, Li A, Prifti E, Chen Y, Shao L, Guo J, Le Chatelier E, Yao J, Wu L (2014). Alterations of the human gut microbiome in liver cirrhosis. Nature.

[10] Cadwell K (2015). The virome in host health and disease. Immunity.

[11] Boulant S, Stanifer M, Lozach P-Y (2015). Dynamics of virus-receptor interactions in virus binding, signaling, and endocytosis. Viruses.

[12] Baranowski E, Ruiz-Jarabo CM, Domingo E (2001). Evolution of cell recognition by viruses. Science.

[13] Casasnovas JM (2013). Virus-receptor interactions and receptor-mediated virus entry into host cells. Subcell Biochem.

[14] Li F (2016). Structure, function, and evolution of coronavirus spike proteins. Ann Rev Virol.

[15] Peng W, de Vries RP, Grant OC, Thompson AJ, McBride R, Tsogtbaatar B, Lee PS, Razi N, Wilson IA, Woods RJ (2017). Recent h3n2 viruses have evolved specificity for extended, branched human-type receptors, conferring potential for increased avidity. Cell Host Microbe.

[16] Zhang Z, Zhu Z, Chen W, Cai Z, Xu B, Tan Z, Wu A, Ge X, Guo X, Tan Z (2018). Cell membrane proteins with high n-glycosylation, high expression and multiple interaction partners are preferred by mammalian viruses as receptors. Bioinformatics.

[17] Laarhoven TV, Nabuurs SB, Marchiori E (2011). Gaussian interaction profile kernels for predicting drugÿtarget interaction. Bioinformatics.

[18] Yan C, Guihua D, Wu FX, Pan Y, Wang J. Brwmda:predicting microbe-disease associations based on similarities and bi-random walk on disease and microbe networks. IEEE/ACM Trans Comput Biol Bioinform. 2019. 10.1109/TCBB.2019.2907626. 10.1109/TCBB.2019.2907626 30932846

[19] Yan C, Wang J, Wu F-X (2018). Dwnn-rls: regularized least squares method for predicting circrna-disease associations. BMC Bioinformatics.

[20] Yan C, Duan G, Wu F, Pan Y, Wang J. Mchmda: Predicting microbe-disease associations based on similarities and low-rank matrix completion. IEEE/ACM Trans Comput Biol Bioinform. 2019. 10.1109/TCBB.2019.2926716. 10.1109/TCBB.2019.2926716 31295117

[21] Lan W, Wang J, Li M, Liu J, Wu F-X, Pan Y (2018). Predicting microrna-disease associations based on improved microrna and disease similarities. IEEE/ACM Trans Comput Biol Bioinform.

[22] Lan W, Li M, Zhao K, Liu J, Wu F-X, Pan Y, Wang J (2016). Ldap: a web server for lncrna-disease association prediction. Bioinformatics.

[23] Kanehisa M, Goto S, Hattori M, Aoki-Kinoshita KF, Itoh M, Kawashima S, Katayama T, Araki M, Hirakawa M (2006). From genomics to chemical genomics: new developments in kegg. Nucleic Acids Res.

[24] Smith TF, Waterman MS (1981). Identification of common molecular subsequences. J Mol Biol.

[25] Jiang H, Wang J, Li M, Lan W, Wu F, Pan Y. mirtrs: A recommendation algorithm for predicting mirna targets. IEEE/ACM Trans Comput Biol Bioinform. 2018. 10.1109/TCBB.2018.2873299. 10.1109/TCBB.2018.2873299 30281478

[26] Xia Z, Wu L-Y, Zhou X, Wong ST (2010). Semi-supervised drug-protein interaction prediction from heterogeneous biological spaces. BMC Syst Biol.

[27] Yuan Q, Gao J, Wu D, Zhang S, Mamitsuka H, Zhu S (2016). Druge-rank: improving drug–target interaction prediction of new candidate drugs or targets by ensemble learning to rank. Bioinformatics.

[28] Yan C, Wang J, Lan W, Wu F-X, Pan Y (2017). Sdtrls: Predicting drug-target interactions for complex diseases based on chemical substructures. Complexity.

[29] Belkin M, Niyogi P, Sindhwani V (2006). Manifold regularization: A geometric framework for learning from labeled and unlabeled examples. J Mach Learn Res.

[30] Luo H, Wang J, Li M, Luo J, Peng X, Wu F-X, Pan Y (2016). Drug repositioning based on comprehensive similarity measures and bi-random walk algorithm. Bioinformatics.

[31] Zheng X, Ding H, Mamitsuka H, Zhu S. Collaborative matrix factorization with multiple similarities for predicting drug-target interactions. In: Proceedings of the 19th ACM SIGKDD International Conference on Knowledge Discovery and Data Mining. ACM: 2013. p. 1025–33. 10.1145/2487575.2487670.

[32] Sakuntabhai A, Turbpaiboon C, Casadémont I, Chuansumrit A, Lowhnoo T, Kajaste-Rudnitski A, Kalayanarooj SM, Tangnararatchakit K, Tangthawornchaikul N, Vasanawathana S (2005). A variant in the cd209 promoter is associated with severity of dengue disease. Nat Genet.

[33] Garcia-Vallejo JJ, van Kooyk Y (2015). Dc-sign: the strange case of dr. jekyll and mr. hyde. Immunity.

[34] Lo AW, Tang NL, To K-F (2006). How the sars coronavirus causes disease: host or organism?. J Pathol J Pathol Soc Great B Irel.

[35] Li H, Wang J-X, Wu D-D, Wang H-W, Tang NL-S, Zhang Y-P (2012). The origin and evolution of variable number tandem repeat of clec4m gene in the global human population. PLoS ONE.

[36] Léger P, Tetard M, Youness B, Cordes N, Rouxel RN, Flamand M, Lozach P-Y (2016). Differential use of the c-type lectins l-sign and dc-sign for phlebovirus endocytosis. Traffic.

[37] Yan C, Wang J, Ni P, Lan W, Wu F, Pan Y (2019). Dnrlmf-mda: Predicting microrna-disease associations based on similarities of micrornas and diseases. IEEE/ACM Trans Comput Biol Bioinform.

[38] Luo H, Li M, Wang S, Liu Q, Li Y, Wang J (2018). Computational drug repositioning using low-rank matrix approximation and randomized algorithms. Bioinformatics.

[39] Lu C, Yang M, Luo F, Wu F-X, Li M, Pan Y, Li Y, Wang J (2018). Prediction of lncrna–disease associations based on inductive matrix completion. Bioinformatics.

[40] Yang M, Luo H, Li Y, Wang J (2019). Drug repositioning based on bounded nuclear norm regularization. Bioinformatics.

